# Draft Genome Sequence of *Gallibacterium anatis* bv. haemolytica 12656-12 Liver, an Isolate Obtained from the Liver of a Septicemic Chicken

**DOI:** 10.1128/genomeA.00810-13

**Published:** 2013-10-10

**Authors:** Egle Kudirkiene, Henrik Christensen, Anders Miki Bojesen

**Affiliations:** Department of Veterinary Disease Biology, Faculty of Health and Medical Sciences, University of Copenhagen, Frederiksberg C, Denmark

## Abstract

We report the draft genome sequence of *Gallibacterium anatis* bv. haemolytica strain 12656-12 Liver. This strain was isolated from the liver of a septicemic layer chicken in Denmark in 1981. The strain has been used extensively for experimental purposes.

## GENOME ANNOUNCEMENT

*Gallibacterium anatis* is a cause of infectious peritonitis and salpingitis in laying hens and breeders, resulting in a significant drop in egg production and in increased mortality (1, 2). *G. anatis* bv. haemolytica strain 12656-12 Liver was isolated from the liver of a septicemic layer chicken in Denmark in 1981. It represents the pathogenic species *G. anatis*, which may cause severe infection in both spontaneously infected (3) and experimentally infected chickens (2). This strain has been used extensively as a reference strain in previous studies addressing pathogenicity, genotyping, and antimicrobial resistance (4–8). Recently, a number of putative antigens identified in a previously published *G. anatis* genome (9), including the recombinant repeats in toxin (RTX)-like toxin GtxA and the F17-like fimbrial subunit protein FlfA, were produced using this strain and were characterized in detail (10–12).

Genomic DNA from 12656-12 Liver was isolated using a blood and tissue kit (catalog no. 69506; Qiagen). The draft genome sequencing was performed at the National High-Throughput DNA Sequencing Center of the University of Copenhagen. The size of the genomic libraries ranged from 200 to 1,000 bp, with an average length of 400 bp (insert size, 250 bp). The MiSeq instrument (Illumina) was used for the genome sequencing at a 150-bp paired-end-read format. The run yielded high-quality filtered 1,297,408 sequences in pairs containing 186,212,739 bases, which provided an average of 70-fold coverage of the genome. All reads were *de novo* assembled using the CLC Genomic Workbench 5.5.1 software package (CLC, Denmark). The assembly revealed 88 contigs of 408 bp to 139,905 bp in size. The prediction of protein-coding sequences and annotation were performed by the NCBI Prokaryotic Genomes Automatic Annotation Pipeline (http://www.ncbi.nlm.nih.gov/genomes/static/Pipeline.html). The total estimated size of the genome is 2,571,587 bp, with a G+C content of 39.5%. The genome contains 2,385 coding DNA sequences (CDSs), 6 rRNAs, and 54 tRNA sequences. A similar G+C content of 40% was reported previously for a published genome sequence of *G. anatis* strain UMN179 (9); however, the latter is larger and has 147 more CDSs than the genome of strain 12656-12 Liver. Importantly, the genes encoding previously identified universal immunogens (9) (A. M. Bojesen, unpublished data) were detected, which allows for the use of this strain in future attempts in vaccine development.

The sequence of 12656-12 Liver will be helpful for future attempts to understand the pathogenic mechanism of *G. anatis* and will contribute to investigations of antigen evolution and regulation.

### Nucleotide sequence accession numbers.

This whole-genome shotgun project has been deposited at DDBJ/EMBL/GenBank under the accession no. AVOX00000000. The version described in this paper is version AVOX01000000.
